# Supplementary material for: Crystal structure prediction by combining graph network and optimization algorithm
Source: Nat Commun. 2022 Mar 21;13:1492. doi: 10.1038/s41467-022-29241-4 (PMC8938491; doi:10.1038/s41467-022-29241-4)
Supplement: Supplementary file 1 — Supplementary Information [file 41467_2022_29241_MOESM1_ESM.pdf]

Supplementary Information

Crystal structure prediction via combining graph network and optimization algorithm

G. Cheng et al.

Supplementary Tables

Supplementary Table 1. The setup pf hyperparameters used in graph network (GN), Bayesian optimization (BO) and Particle-swarm optimization (PSO).

Algorithm	Hyperparameter	Value
GN	Size of embedding layer, N_v	16
	Number of points from the expanded distance with Gaussian basis, N_e	100
	Number of MEGNet layers, m	3
	Size of a MEGNet layer	(64, 64, 32)
	Size of set2set layer	16
	Number of dense layers, l	2
	Size of dense layers	[32, 16]
BO	γ	0.25
	Number of init points	200
	Max steps	5000
PSO	ω	0.8
	c_p	0.5
	c_g	0.5
	Number of particles	200
	Max iterations	25

Supplementary Figures

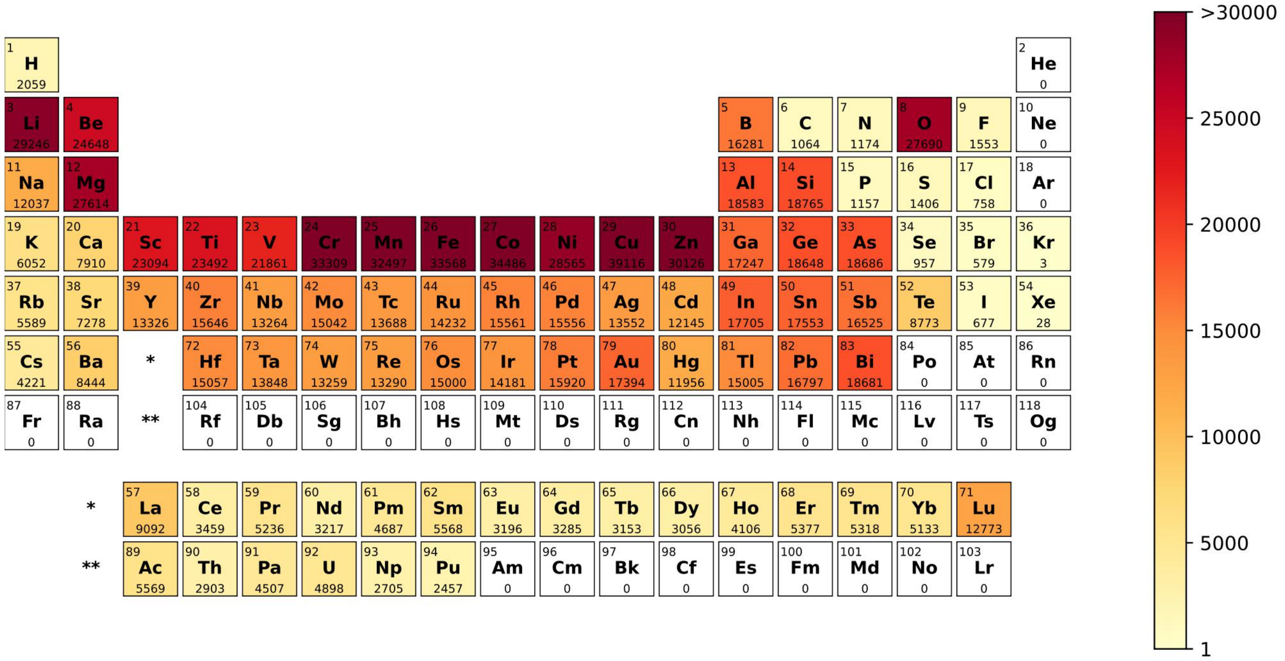

Supplementary Figure 1. The number of elemental appearance in OQMD.

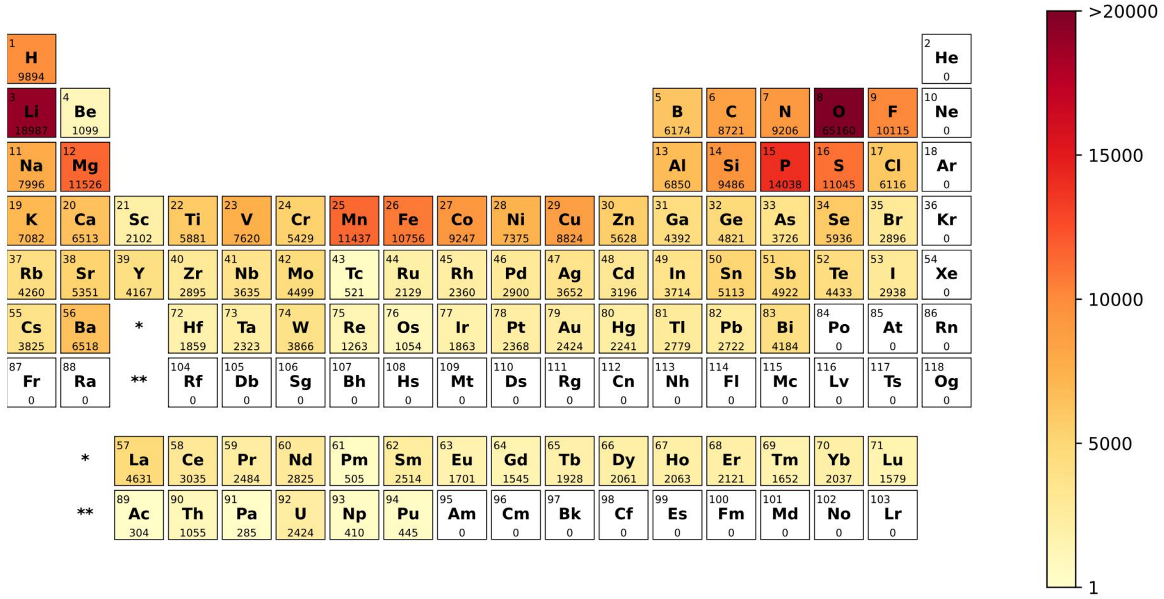

Supplementary Figure 2. The number of elemental appearance in MatB.

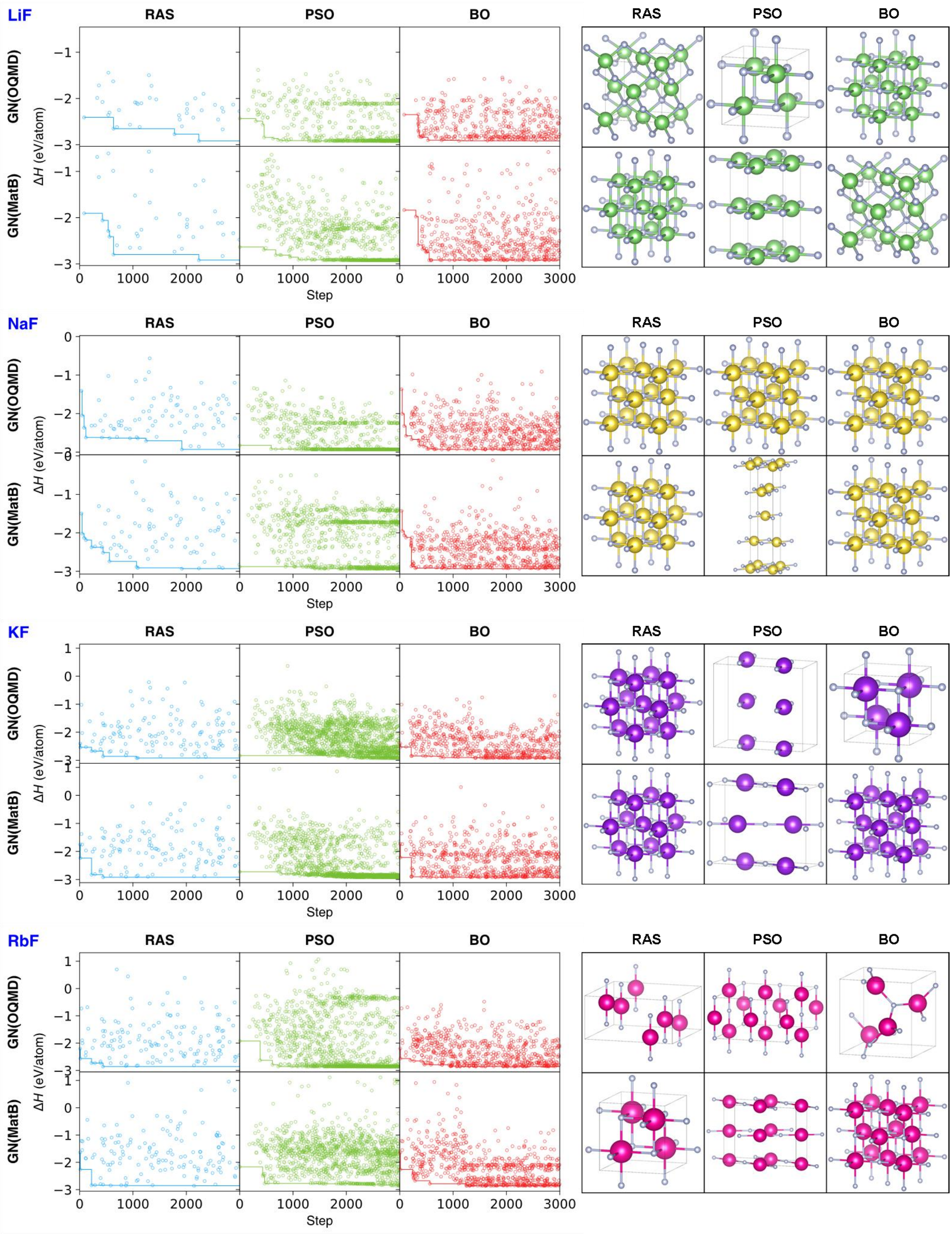

Supplementary Figure 3. The process of GN(OQMD)-RAS, GN(OQMD)-PSO, GN(OQMD)-BO, GN(MatB)-RAS, GN(MatB)-PSO and GN(MatB)-BO approaches to search the crystal structures of LiF, NaF, KF, and RbF. Lowest-energy structures within 3,000 steps for each approach have been shown at right side.

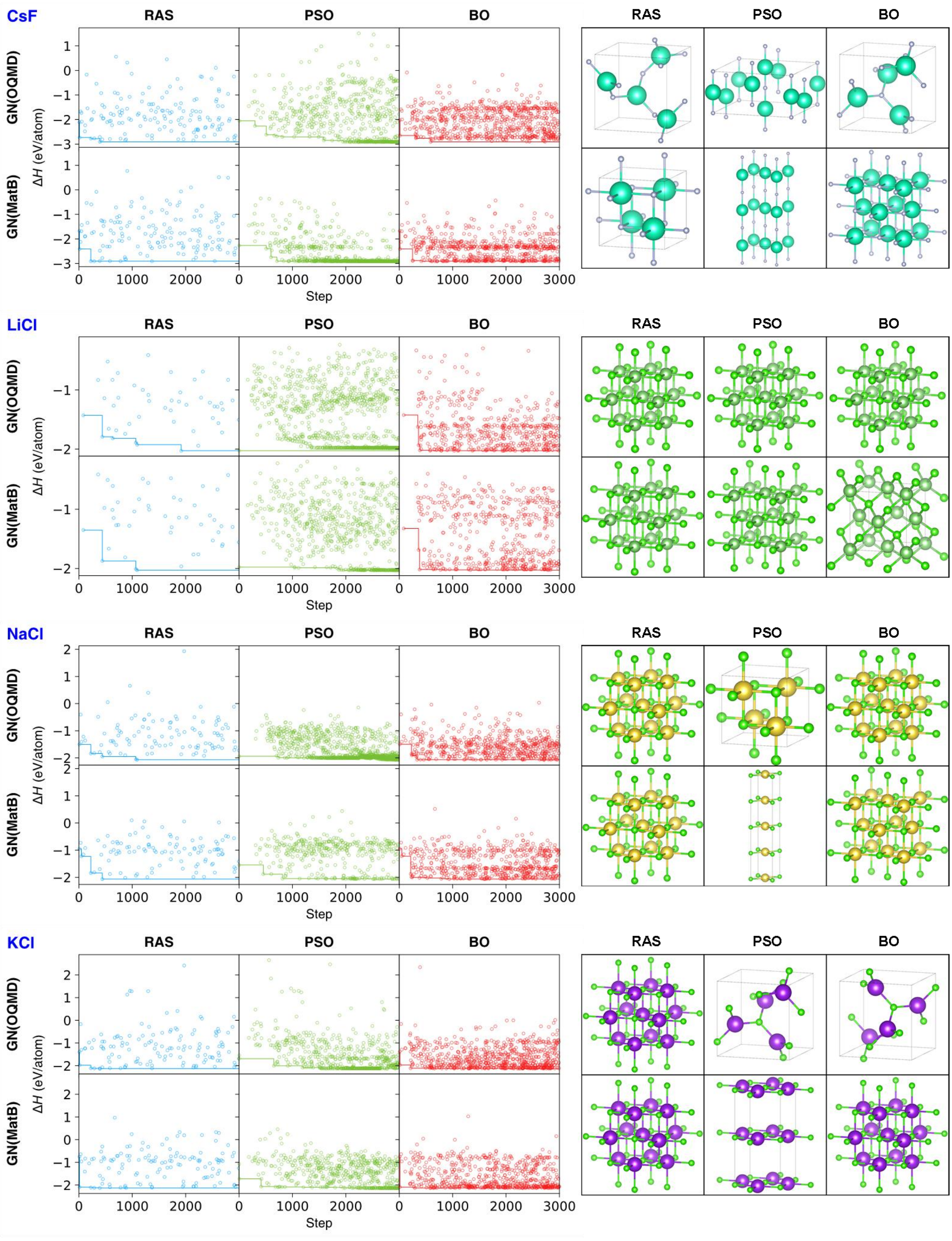

Supplementary Figure 4. The process of GN(OQMD)-RAS, GN(OQMD)-PSO, GN(OQMD)-BO, GN(MatB)-RAS, GN(MatB)-PSO and GN(MatB)-BO approaches to search the crystal structures of CsF, LiCl, NaCl, and KCl. Lowest-energy structures within 3,000 steps for each approach have been shown at right side.

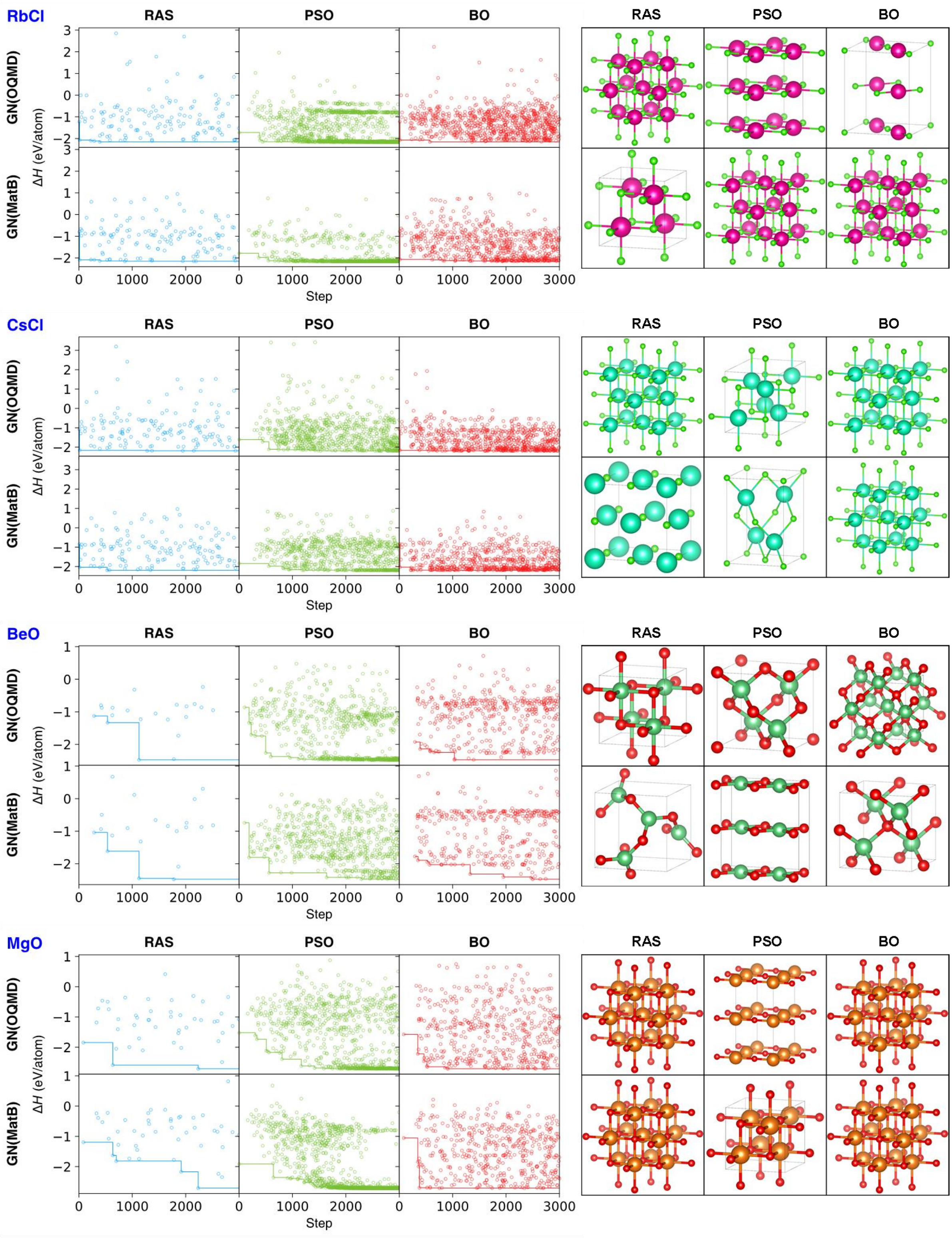

Supplementary Figure 5. The process of GN(OQMD)-RAS, GN(OQMD)-PSO, GN(OQMD)-BO, GN(MatB)-RAS, GN(MatB)-PSO and GN(MatB)-BO approaches to search the crystal structures of RbCl, CsCl, BeO, and MgO. Lowest-energy structures within 3,000 steps for each approach have been shown at right side.

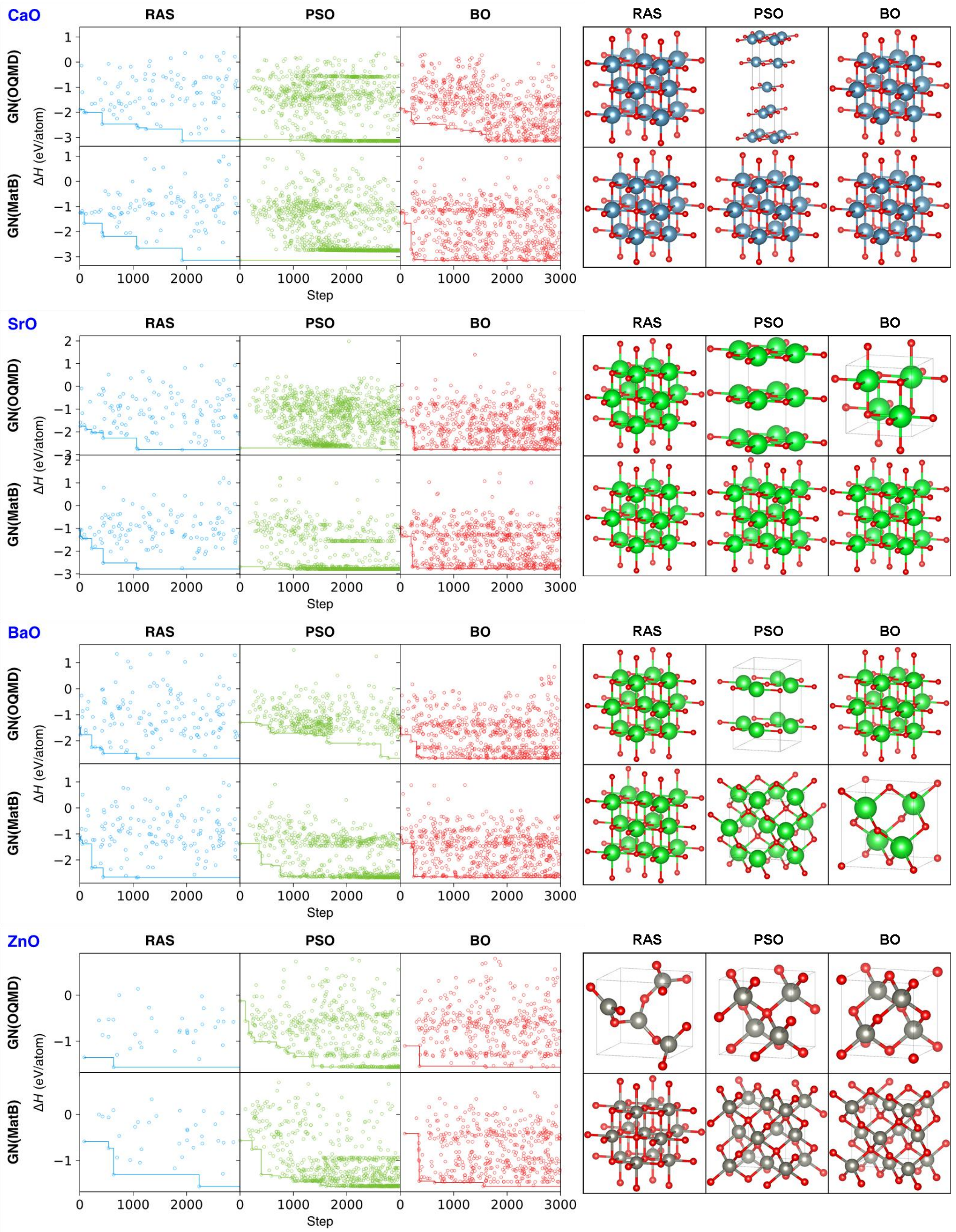

Supplementary Figure 6. The process of GN(OQMD)-RAS, GN(OQMD)-PSO, GN(OQMD)-BO, GN(MatB)-RAS, GN(MatB)-PSO and GN(MatB)-BO approaches to search the crystal structures of CaO, SrO, BaO, and ZnO. Lowest-energy structures within 3,000 steps for each approach have been shown at right side.

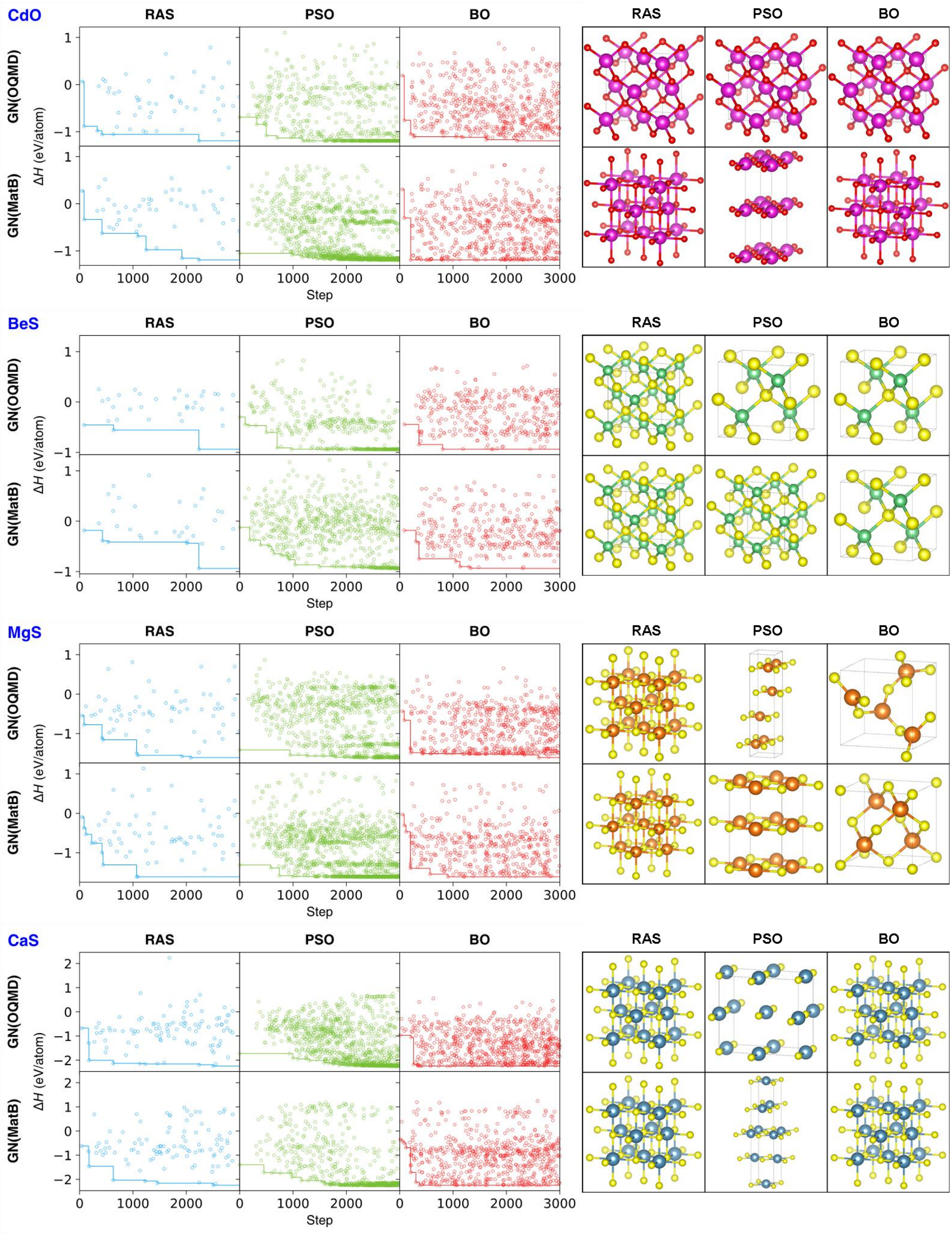

Supplementary Figure 7. The process of GN(OQMD)-RAS, GN(OQMD)-PSO, GN(OQMD)-BO, GN(MatB)-RAS, GN(MatB)-PSO and GN(MatB)-BO approaches to search the crystal structures of CdO, BeS, MgS, and CaS. Lowest-energy structures within 3,000 steps for each approach have been shown at right side.

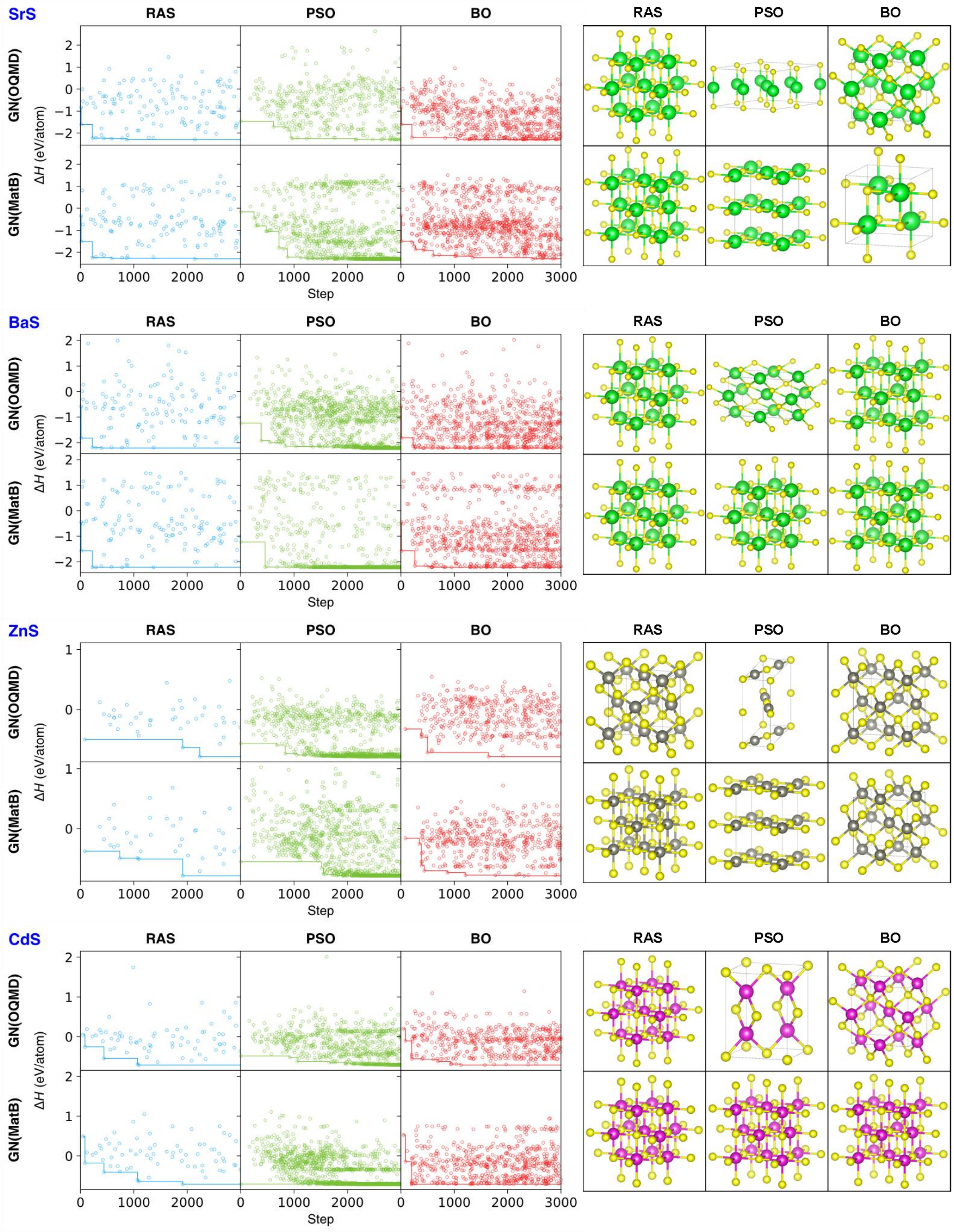

Supplementary Figure 8. The process of GN(OQMD)-RAS, GN(OQMD)-PSO, GN(OQMD)-BO, GN(MatB)-RAS, GN(MatB)-PSO and GN(MatB)-BO approaches to search the crystal structures of SrS, BaS, ZnS, and CdS. Lowest-energy structures within 3,000 steps for each approach have been shown at right side.

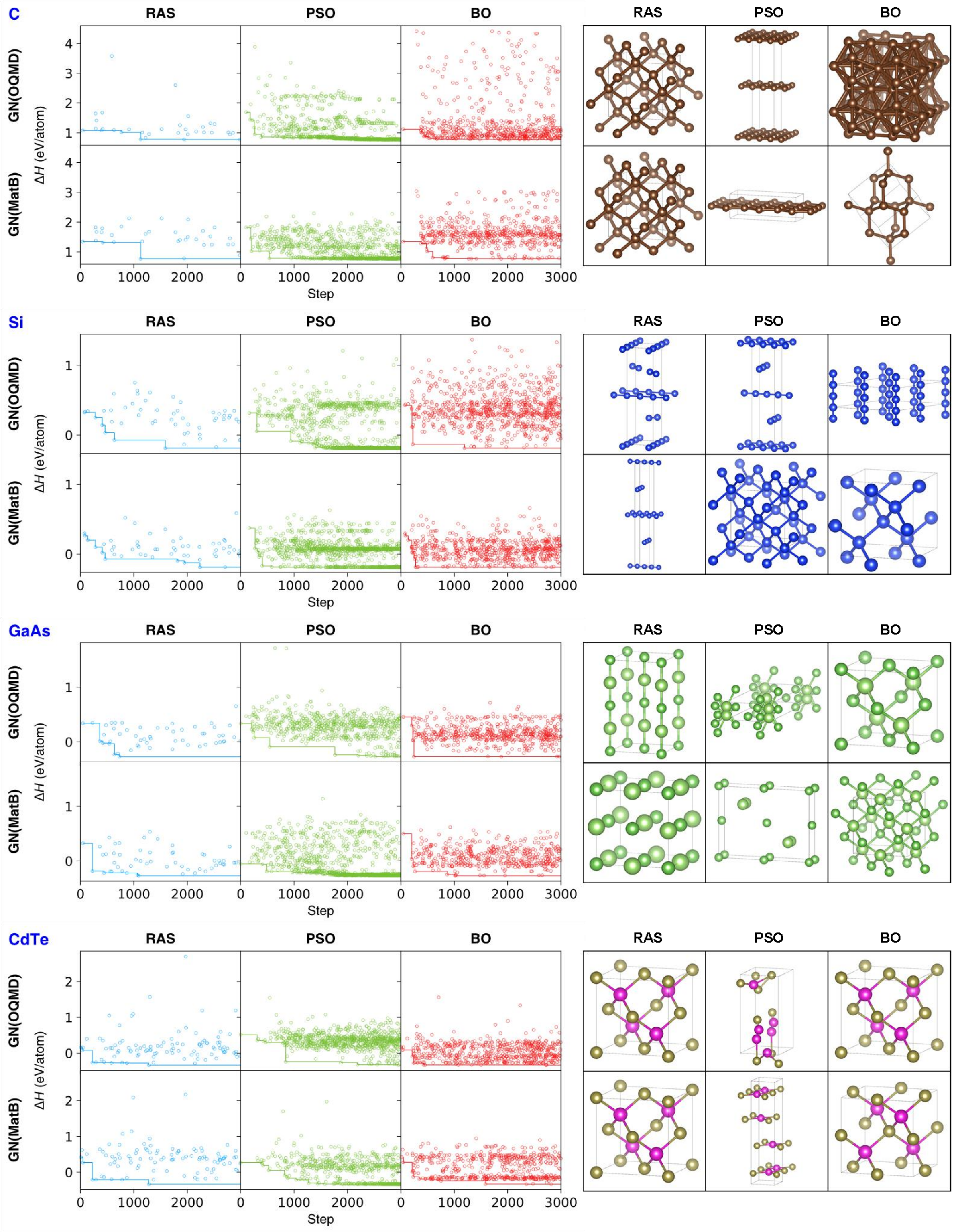

Supplementary Figure 9. The process of GN(OQMD)-RAS, GN(OQMD)-PSO, GN(OQMD)-BO, GN(MatB)-RAS, GN(MatB)-PSO and GN(MatB)-BO approaches to search the crystal structures of C, Si, GaAs, and CdTe. Lowest-energy structures within 3,000 steps for each approach have been shown at right side.

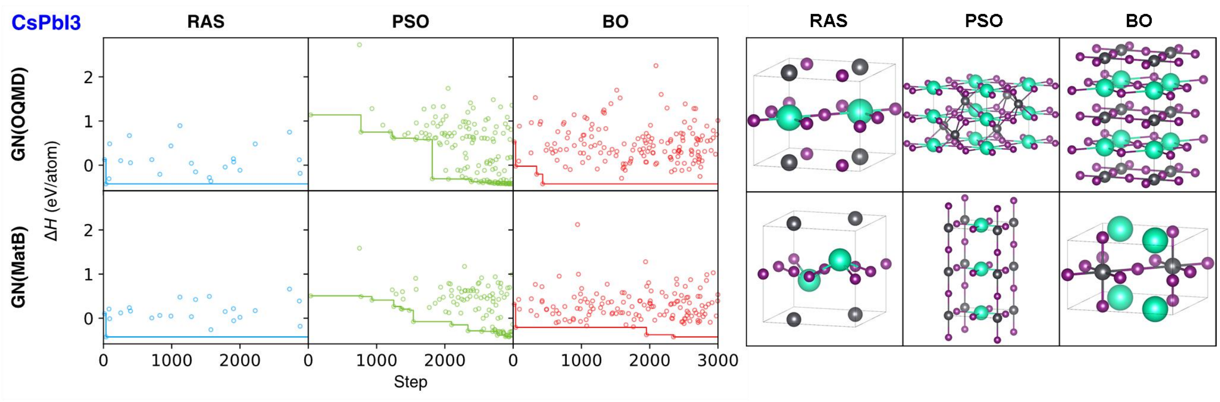

Supplementary Figure 10. The process of GN(OQMD)-RAS, GN(OQMD)-PSO, GN(OQMD)-BO, GN(MatB)-RAS, GN(MatB)-PSO and GN(MatB)-BO approaches to search the crystal structures of $CsPbI_3$. Lowest-energy structures within 3,000 steps for each approach have been shown at right side.

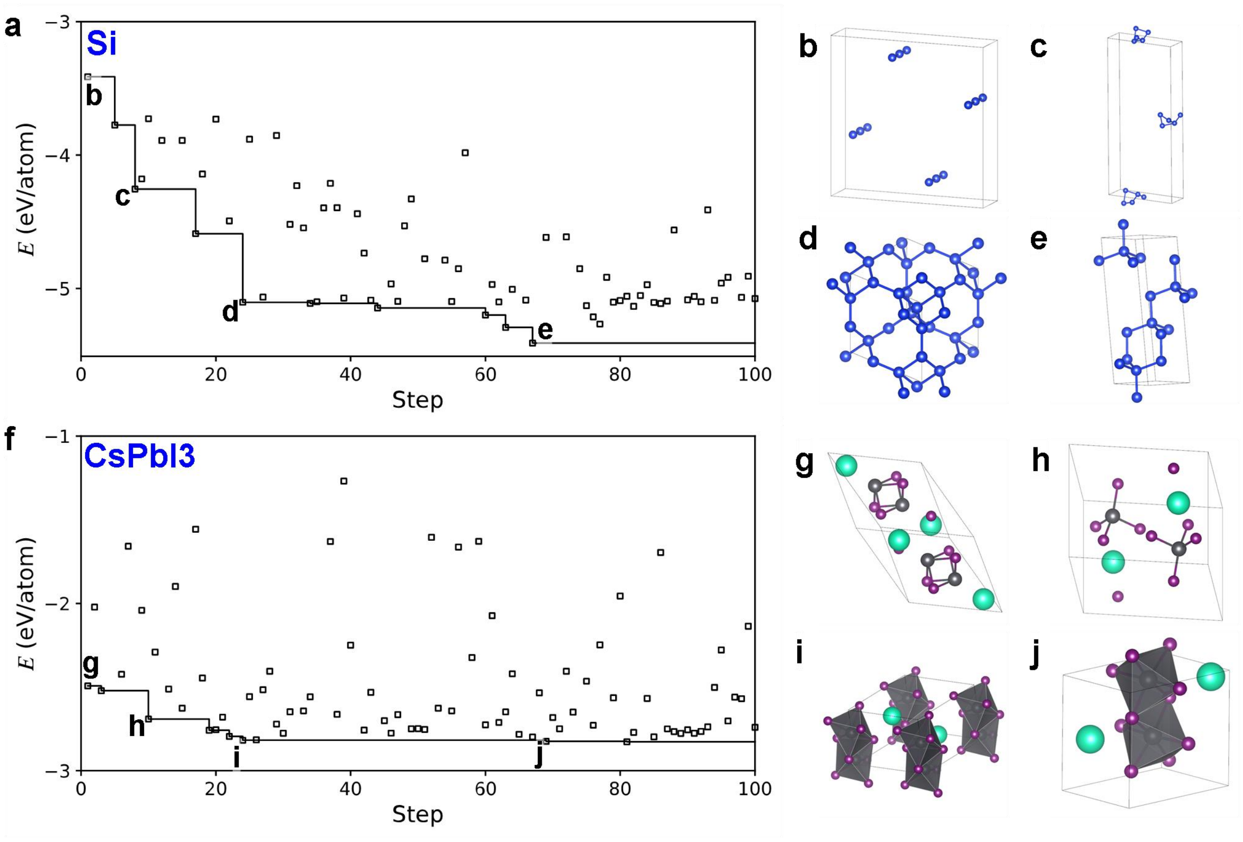

Supplementary Figure 11. The process of DFT-PSO approaches to search the crystal structures of Si and $CsPbI_3$.

CdS

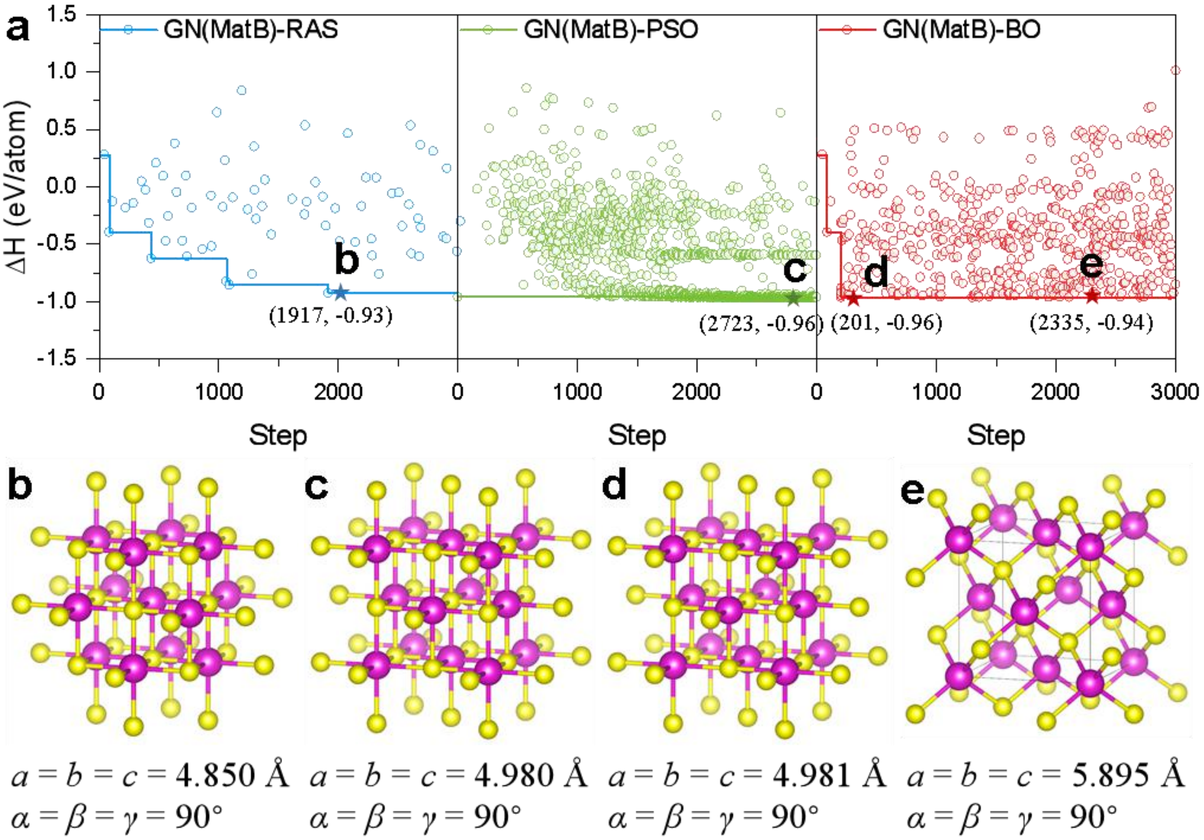

Supplementary Figure 12. (a) Process of GN(MatB)-RAS, GN(MatB)-PSO, and GN(MatB)-BO to search the GSS of CdS. The corresponding structures have been shown in (b-e).

CaS

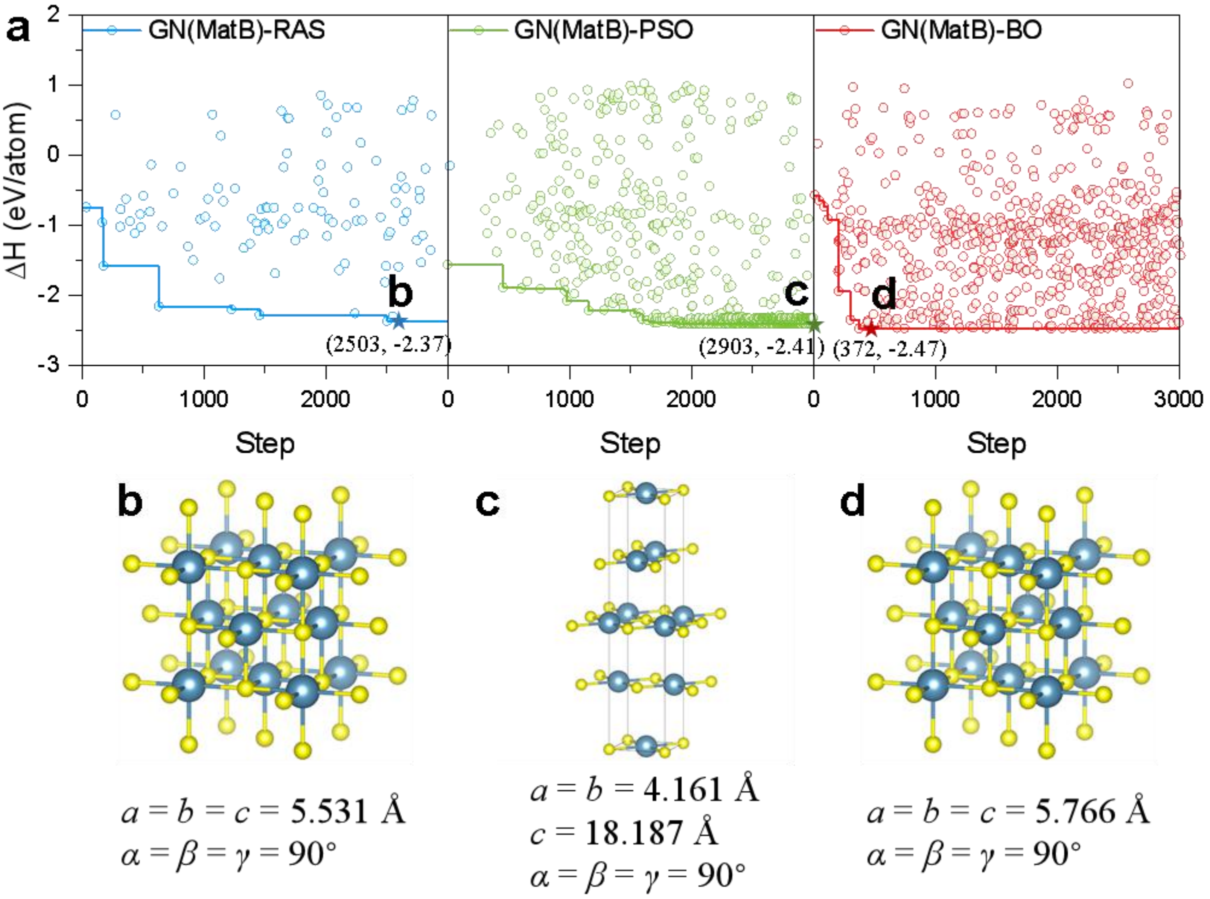

Supplementary Figure 13. (a) Process of GN(MatB)-RAS, GN(MatB)-PSO, and GN(MatB)-BO to search the GSS of CaS. The corresponding structure have been shown in (b-d).
